# Reliability of measures of impairments associated with patellofemoral pain syndrome

**DOI:** 10.1186/1471-2474-7-33

**Published:** 2006-03-31

**Authors:** Sara R Piva, Kelley Fitzgerald, James J Irrgang, Scott Jones, Benjamin R Hando, David A Browder, John D Childs

**Affiliations:** 1 Department of Physical Therapy, School of Health and Rehabilitation Sciences, University of Pittsburgh, USA; 2 Ramstein Outpatient Physical Medicine Clinic, Ramstein Air Force Base, Germany; 3 Eglin Regional Hospital, Eglin Air Force Base, FL, USA; 4 Wilford Hall Medical Center, Lackland Air Force Base, TX, USA; 5 US Army-Baylor University Doctoral Program in Physical Therapy, USA

## Abstract

**Background:**

The reliability and measurement error of several impairment measures used during the clinical examination of patients with patellofemoral pain syndrome (PFPS) has not been established. The purpose was to determine the inter-tester reliability and measurement error of measures of impairments associated with PFPS in patients with PFPS.

**Methods:**

A single group repeated measures design was used. Two pairs of physical therapists participated in data collection. Examiners were blinded to each others' measurements.

**Results:**

Thirty patients (age 29 +/- 8; 17 female) with PFPS participated in this study. Inter-tester reliability coefficients were substantial for measures of hamstrings, quadriceps, plantarflexors, and ITB/TFL complex length, hip abductors strength, and foot pronation (ICCs from .85 to .97); moderate for measures of Q-angle, tibial torsion, hip external rotation strength, lateral retinacular tightness, and quality of movement during a step down task (ICCs from .67 to .79); and poor for femoral anteversion (ICC of .45). Standard error of measurement (SEM) for measures of muscle length ranged from 1.6 degrees to 4.3 degrees. SEM for Q-angle, tibial torsion, and femoral anteversion were 2.4 degrees, 2.9 degrees, and 4.5 degrees respectively. SEM for foot pronation was 1 mm. SEM for measures of muscle strength was 1.8 Kg for abduction and 2.4 Kg for external rotation.

**Conclusion:**

Several of the impairments associated with PFPS had sufficient reliability and low measurement error. Further investigation is needed to test if these impairment measurements are related to physical function and whether or not they are useful for decision-making.

## Background

Patellofemoral pain syndrome (PFPS) is a common knee problem among young active individuals [1-3]. The mechanism of PFPS is not well understood. It has been proposed that PFPS may arise from abnormal muscular and biomechanical factors that alter tracking of the patella within the femoral trochlear notch contributing to increased patellofemoral contact pressures that result in pain and dysfunction [4,5]. Authors have suggested a variety of impairments involved in the etiology of PFPS [6-8]. However, there is no evidence that these impairments are associated with the patient's functional limitations. In the absence of definitive impairments in which to focus the examination or treatment in patients with PFPS, clinicians tend to perform an extensive physical examination that generally includes a multitude of impairment measures such as muscle weakness, soft tissue tightness, structural or postural alterations, and poor quality of movement [3].

Reliability and measurement error are essential properties of any measurement that need to be established before the measurement can be considered clinically meaningful and useful. Reliability is the ability of a test to consistently yield more or less the same results when administered on several occasions to stable subjects, whereas measurement error provides the threshold for interpreting test results being reasonably confident that true change has occurred [9,10]. Although several studies have investigated the reliability of impairment measures associated with patellofemoral dysfunction in healthy subjects [11-15], the reliability and measurement error of impairment measures used during the clinical examination of patients with PFPS has not been established.

Among the measures of muscle strength performed in patients with PFPS, reliability of hip abduction and hip external rotation strength tests have not been determined in patients with PFPS. Hip abductor and external rotation strength are commonly measured in patients with PFPS because weakness of these muscles has been linked with PFPS [16,17]. Authors have suggested these muscles help to maintain pelvic stability by eccentrically controlling femoral internal rotation during weight-bearing activities. Weakness may result in increased medial femoral rotation and valgus knee moments, augmenting compressive forces on the patellofemoral joint [16,17]. Ireland et al [18] suggest that individuals with PFPS have weaker hip muscles when compared to matched control groups. Another study has shown that hip abduction strength is one of the variables able to distinguish between patients with and without PFPS [19].

Soft tissue restrictions, such as shortening of the quadriceps, hamstrings, and plantarflexor muscles, shortening of the iliotibial band/tensor fascia lata (ITB/TFL) complex, and shortening of the lateral retinacular structures have all been associated with PFPS and are impairments commonly measured in this population [20-22]. It is theorized that tight quadriceps and hamstrings may increase compression of the patellofemoral joint [20]. While two studies agree supporting the association of quadriceps flexibility and PFPS, the same studies conflict regarding the association of hamstrings flexibility and PFPS [21,22]. There is some evidence to support the association between plantar flexors tightness and PFPS [21]. Concerning the ITB/TFL and lateral retinacular tissues, although it has been theorized that tightness of these tissues may displace the patella laterally and increase the stress in the patellofemoral joint or medial retinacular tissue [1,23], evidence to support such theory does not yet exist. In general, studies investigating the measurement properties of the above mentioned soft tissue measures have not used individuals with PFPS, or have not determined the measurement error [11,12,24-30].

Studies examining the measurement properties of tests used to determine structural or postural alterations in patients with PFPS are also lacking. Some structural or postural alterations that have been linked to PFPS are excessive foot pronation, quadriceps angle (Q-angle), tibial torsion, and femoral anteversion. Evidence to support that increased foot pronation causes PFPS is inconclusive [6,31]. Regarding Q-angle, it was reported that Q-angle is more accentuated in runners with PFPS than in runners without PFPS [7]. To our knowledge, just one study has investigated the relationship between tibial torsion and PFPS and reported that the lateral rotation of the tibia relative to the femur was increased in patients with PFPS [32]. Studies that investigated the association of femoral anteversion and PFPS have reported conflicting results [32,33]. Although some measures of structural alterations have shown good reliability [14], samples of patients with PFPS have rarely been used [13-15]. A recent study using patients with PFPS reported poor consistency of these measurements [30].

Quality of movement, sometimes referred to as neuromotor control or movement coordination, refers to the biomechanics of the lower extremities, trunk and arms in relationship with its surrounding during physical activities [4]. It has been theorized that patients with PFPS exhibit altered movement patterns in the lower extremities that may result in alterations of the load distribution across the patellofemoral joint [1,21,34]. Altered movement patterns may be recognized during physical activities as movements performed with poor quality. We are unaware of studies that investigated the consistency of measures of quality of movement in patients with PFPS.

The purpose of this study was to determine the inter-tester reliability and measurement error of measures of impairments associated with PFPS in a population of patients diagnosed with PFPS. We have selected to examine the measurement properties of measures of hip abduction strength, hip external rotation strength, quadriceps length, hamstrings length, plantar flexors length, ITB/TFL complex length, lateral retinacular structures length, foot pronation, Q-angle, tibial torsion, femoral anteversion and quality of movement, because of their frequent use in the examination of individuals with PFPS and the lack of information concerning their reliability and measurement error.

## Methods

A single group repeated measures design was used in this study. Data for this study was obtained as part of a larger multicenter study that investigated predictors of function in persons with PFPS.

### Subjects

Individuals were eligible to participate in this study if they were diagnosed by a physician with PFPS, were between 12 and 50 years of age, had pain in one or both knees, had duration of signs and symptoms greater than 4 weeks, had history of insidious onset not related to trauma, and had pain in the patellar region with at least three of the following: manual compression of the patella against the femur at rest or during an isometric knee extensor contraction, palpation of the postero-medial and postero-lateral borders of the patella, resisted isometric quadriceps femoris muscle contraction, squatting, stair climbing, kneeling, or prolonged sitting.

Exclusion criteria included previous patellar dislocation, knee surgery over the past 2 years, concomitant known or suspected diagnosis of: peripatellar bursitis or tendonitis, internal knee derangement, systemic arthritis, ligamentous knee injury or laxity, plica syndrome, Sinding-Larsen-Johansson's disease, Osgood Schlatter's disease, infection, malignancy, musculoskeletal or neurological lower extremity involvement that interferes with physical activity, and pregnancy. Thirty patients were recruited from 2 clinical sites: Wilford Hall Medical Center, in San Antonio, TX, and University of Pittsburgh's Centers for Rehab Services, Pittsburgh, PA). All subjects who agreed to participate signed a consent form approved by the Institutional Review Board of the respective clinical site. Demographic characteristics of the participants are reported in Table 1.

### Procedures

Subjects had one lower extremity tested unless they had bilateral symptoms, in which case the most symptomatic side was tested. The most symptomatic knee was determined by the patient's self-report. Data were collected during one assessment session that lasted approximately 60 minutes. We collected data during the same assessment session to ensure the subjects remained as stable as possible (did not change) in the parameters tested. Examiners met once during a 2-hour session before the study was initiated to review operational definitions and practice the procedures to ensure standardization. Each examiner was provided with the Manual of Standard Operating Procedures of the study, which contained detailed explanations about the performance of each test.

Two pairs of physical therapists (1 pair from each site) with different levels of experience participated in data collection. One pair of testers had 3 and 5 years of clinical practice (pair 1); whereas the other pair had 2 and 10 years of clinical experience (pair 2). During each data collection session, the subject remained inside an examination room. To ensure that the examiners remained blinded to each other's assessments, the two examiners entered the examination room independently, performed and recorded the measurements, and then left the room. The results were not shared with the other examiner. The measurements were always performed in the same order. Order of testing was based on patient positioning in the following order: supine, prone, side-lying, and standing positions. This was done to avoid excessive changing of positions, ensure that the examiners were performing all tests under the same conditions, and ensure that any order effect would be the same for each examiner. Each examiner in the pair alternated serving as the initial examiner.

### Measures

Each participant completed a demographic questionnaire and self-reported measures of pain and function prior to the physical examination. Subjects' age, gender, height, weight, prior history of knee problems, mechanism of injury, current episode duration, and symptom location were recorded.

Pain intensity was measured using an 11-point numeric pain rating scale ranging from 0 (No Pain) to 10 (Worst Imaginable Pain). Patients rated their current, best, and worst level of pain during the last 24 hours. The average of the three ratings was used to represent the patient's overall pain intensity. Numeric pain scales have been shown to be reliable and valid [35-38].

The Activity of Daily Living Scale (ADLS) of the Knee Outcome Survey was used as a knee-specific measure of physical function [39]. The ADLS assesses the effects of knee impairment on activities of daily living. The ADLS consists of 14 items that measure the full spectrum of symptoms and functional limitations during activities of daily living that one may experience as a result of a variety of knee pathologies. The ADLS score is transformed to a 0 to 100 point scale with 100 indicating the absence of symptoms and functional limitations. Psychometric testing has demonstrated the ADLS to be reliable, valid and responsive in subjects with PFPS [39,40].

**Figure 1:**
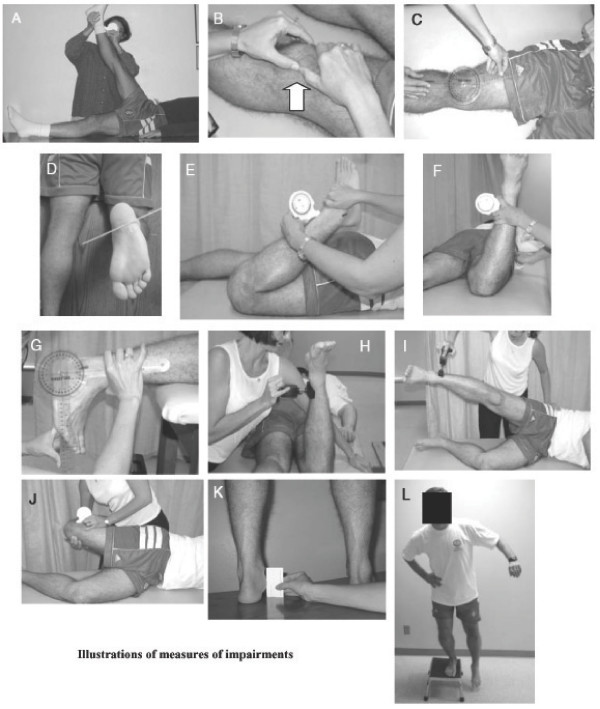
Illustration of the techniques used to measure impairments associated with PFPS (A) Hamstrings length - straight leg raise test (B) Tightness of the lateral retinacular structures - patellar tilt test (C) Q-angle (D) Tibial torsion - angle formed between inter-epicondilar and intermalleolar lines (E) Quadriceps muscle length - quadriceps femoris muscle angle (F) Femoral anteversion -  Craig’s test (G) Plantar flexors length (H) Hip external rotation strength (I) Hip abduction strength (J) ITB/TFL complex length- Ober’s test (K) Foot pronation - navicular drop test (L) Quality of movement - example of lateral step down test trial using arm strategy

Measurements performed during the physical examination were as follows:

**Hamstrings length **was determined by measuring the straight leg raise using a gravity goniometer (MIE Medical Research Ltd., Leeks, UK). The subject was in the supine position with the tested knee extended and the other leg flat on the table to avoid excessive posterior pelvic tilt. Before starting the measurement, the goniometer was zeroed on the lower half of the anterior border of the tibia. Then, the lower extremity was passively lifted to the end range of motion or firm end feel and the measurement recorded in degrees (Figure 1). The average measurement of two trials with 5-second pause between trials was recorded.

**Tightness of the lateral retinacular structures **was assessed with the patellar tilt test [44]. The patellar tilt test was performed with the subject in supine with the knee in full extension and the femoral condyles placed in the horizontal plane. The examiner attempted to lift the lateral edge of the patella from the lateral femoral condyle. The patella was not allowed to move laterally during the measurement (Figure 1). The inability to lift the lateral boarder of the patella above the horizontal plane indicated a positive test for tightness of the lateral retinaculum. Adequate length of the lateral retinaculum or negative test was indicated by the ability to lift the lateral boarder of the patella above the horizontal plane. Tightness of lateral retinaculum was scored as tight or normal.

**Q-Angle **was measured with the knee in full extension with the subject supine. The angle formed by the intersection of the line of application of the quadriceps force (line from the anterior superior iliac spine to the center of patella) with the center line of the patellar tendon (line from the center of the patella to the tibial tubercle) was measured in degrees with a universal goniometer (Figure 1) [42]. The center of the patella and the tibial tubercle were marked with a demographic pencil, which was wiped out after the measurement. Before the measurement the tester palpated the anterior superior iliac spine and asked the subject to keep his second finger pointing down over this landmark during the measurement. Subject was also asked not to contract the quadriceps muscles during the measurement.

**Tibial torsion **was measured with a universal goniometer with the participant prone on a low table, and with the tested knee bent at 90°. Height of the table was adjusted so the tester could comfortably visualize the plantar surface of the subject's foot. To facilitate visualization, the tester marked the most prominent aspect of the medial and lateral malleolus with a small dot. The examiner measured the angle formed by the axis of the knee (imaginary line from the medial to lateral femoral epicondile) and an imaginary line through the malleoli (Figure 1). We elected to measure tibial torsion with the patient in a prone position rather than the position usually described with the patient sitting with knees in 90° because tibial torsion is a horizontal plane rotational malalignment [43,44]. We believe using an inferior view of the leg enables better observation of the talocrural joint axis in the horizontal plane.

**Quadriceps length **was determined by measuring the quadriceps femoris muscle angle during passive knee flexion with the subject in the prone position. Care was taken to avoid anterior tilting of the pelvis and/or extension of the lumbar spine. The angle of knee flexion in the prone position was measured using a gravity goniometer which was zeroed on a horizontal surface prior to the measurements. The gravity goniometer was placed over the distal tibia (Figure 1). The average measurement of two trials with 5-second pause between trials was recorded.

**Femoral anteversion **was measured using the Craig's test with the participant in the prone position with the knee flexed to 90° [45]. Before starting the measurement, the gravity goniometer was zeroed on a vertical surface and placed on the medial surface of the lower leg, just proximal to the medial malleolus (Table 2). The examiner palpated the posterior aspect of the greater trochanter of the femur. The hip was then passively rotated until the most prominent portion of the greater trochanter reached the horizontal plane. The degree of anteversion was then estimated based on the angle of the lower leg with the vertical (Figure 1).

**Plantar flexors length **was determined by measuring the amount of ankle joint dorsiflexion with the knee extended and again with the knee flexed at 90°. Ankle dorsiflexion measured with the knee extended was used to account for the influence of gastrocnemius tightness. Measurement of ankle dorsiflexion with the knee bent was used to detect tightness of joint capsule or soleus muscle. The subject was positioned in the prone position with the foot hanging off the table and the subtalar joint was maintained in the neutral position. Dorsiflexion was measured with a standard goniometer as the angle formed by the lateral midline of the leg on a line from the head of the fibula to the tip of the lateral malleolus and the lateral midline of the foot in line with the border of the rearfoot/calcaneus (Figure 1). The average measurement of two trials with 5-second pause between trials was recorded.

**Hip external rotation strength **– Strength measures were performed using the Lafayette Manual Muscle Test (MMT) System (Lafayette Instrument, Lafayette, IN). Muscle strength was recorded in terms of force, in kilograms. Hip external rotation strength was examined with the subject positioned in prone on a padded table with the test knee flexed to 90° and the hip in neutral rotation. The contralateral lower extremity was positioned with the hip in neutral rotation and the knee in full extension. To obtain optimal mechanical advantage, the examiner stood on the side of the table opposite of the test limb. Subjects exerted an isometric contraction of their hip external rotators for 3–5 seconds in a position of neutral hip rotation. The manual resistance against the external rotation was applied with the MMT just proximal to the medial malleolus (Figure 1). To maintain uniformity in the nature of verbal commands provided by the tester during testing, the testers were instructed to always give a strong verbal encouragement during the performance of every maximum effort. The average force of two trials with one minute of rest between trials was recorded.

**Hip abduction strength **was measured with the subject in side-lying with the test hip positioned superior with respect to the contralateral hip. Subjects exerted an isometric contraction of their hip abductors for 3–5 seconds in a position of approximately 30° of hip abduction and 5° of hip extension. The manual resistance was applied with the MMT just proximal to the lateral malleolus in the direction of adduction (Figure 1). To maintain uniformity in the nature of verbal commands provided by the tester during testing, the testers were instructed to always give a strong verbal encouragement during the performance of every maximum effort. The average force of two trials with one minute of rest between trials was recorded.

**Length of the Iliotibial Band/Tensor Fascia Lata (ITB/TFL) Complex **was examined using the Ober's test [46]. The subject was positioned in side-lying with the tested leg positioned superior and the lower leg slightly flexed at the hip and knee to maintain stability. The gravity goniometer was zeroed on a horizontal surface prior to the measurement and was placed over the distal portion of the ITB/TFL complex (Figure 1). The test leg was flexed to a right angle at the knee and grasped just below the knee with the examiner's distal hand. The examiner moved the subject's thigh first in flexion, then through abduction combined with extension until the hip was positioned in mid-range abduction with neutral flexion/extension. From this position the thigh was allowed to drop toward the table until the point where the limb stopped moving toward the table. At that point the measurement was taken. The result was recorded as a continuous variable. Negative values represent more tightness whereas positive values (below horizontal) represent less tightness. The average measurement of two trials with 5-second pause between trials was recorded.

**Foot pronation **was measured by the navicular drop test [14,47]. Navicular drop test measures the difference between height of the navicular at subtalar joint neutral position and that of the relaxed stance position [14,47]. The subject stood on a high hard surface with his feet shoulder width apart. The examiner stayed behind the subject with the eyes leveled at subject's feet. The examiner marked the subject's navicular tuberosity with a demographic pencil, which was wiped out after the measurement. The examiner put the subject in the subtalar joint neutral position. Using an index card placed perpendicular to the table, the examiner recorded the distance from the navicular to the floor (Figure 1). The subject was then instructed to relax from the subtalar neutral position and the measurement was repeated. Then, with a metric ruler, the distance between the two dots, in the index card (which represents the difference in the position of the navicular tubercle with respect to the floor between the subtalar neutral and relaxed standing positions) was recorded in millimeters. Greater distances between the dots indicate greater pronation.

**Quality of movement during the lateral step down test **was assessed using a scale designed for this purpose. The subject was asked to stand in single limb support with the hands on the waist, the knee straight and the foot positioned close to the edge of a 20 cm high step. The contralateral leg was positioned over the floor adjacent to the step and was maintained with the knee in extension. The subject then bent the tested knee until the contralateral leg gently contacted the floor and then re-extended the knee to the start position. This maneuver was repeated for 5 repetitions. The examiner faced the subject and scored the test based on 5 criteria: 1) Arm strategy. If subject used an arm strategy in an attempt to recover balance, 1 point was added (Figure 1); 2) Trunk movement. If the trunk leaned to any side, 1 point was added; 3) Pelvis plane. If pelvis rotated or elevated one side compared with the other, 1 point was added; 4) Knee position. If the knee deviated medially and the tibial tuberosity crossed an imaginary vertical line over the 2^nd ^toe, add 1 point, or, if the knee deviated medially and the tibial tuberosity crossed an imaginary vertical line over the medial border of the foot, add 2 points, and; 5) Maintain steady unilateral stance. If the subject stepped down on the non-tested side, or if the subject tested limb became unsteady (i.e. wavered from side to side on the tested side), add 1 point. Total score of 0 or 1 was classified as good quality of movement, total score of 2 or 3 was classified as medium quality, and total score of 4 or above was classified as poor quality of movement.

### Data analysis

Descriptive statistics, including frequency counts for categorical variables and measures of central tendency and dispersion for continuous variables were calculated to summarize the data. Kolmogorov-Smirnov Z-tests were performed to assess whether continuous data approximated a normal distribution. Inter-tester reliability for categorical or ordinal impairment measurements was determined by a Cohen's Kappa statistics and its 95% CI [48]. Intraclass correlation coefficients (ICC) and their 95% CI were calculated for continuous measures [49,50]. The ICC model (2, 1) was used when the unit of analysis was a single measurement, and the model (2, 2) was used when the unit of analysis represented the mean of 2 ratings [49,50]. The mean square estimates to calculate the ICC coefficients were obtained from a random effects 2-way analysis of variance with repeated measures [50].

Calculation of the standard error of measurement (SEM) was used to determine measurement error. Results of the reliability analyses for the continuous measures were used to calculate the SEM. The SEM was calculated as (SD * v 1 - r), where r is the test-retest reliability coefficient and SD is the standard deviation of the combined scores [51,52].

The sample size was calculated a priori using SamplePower™ (Chicago, Illinois) statistical software based on the calculation of Cohen Kappa coefficients on a dichotomous variable (i.e. tight or not tight during the patellar tilt test). To ensure sufficient statistical power to achieve a lower bound of the 95% confidence interval for Kappa of 0.30, assuming Kappa would be equal to 0.60, a sample size of 30 subjects was needed [48]. This sample size would also be adequate to calculate ICC coefficients on the continuous variables, given that we had 2 testers per subject, hoping for an ICC of .85, and having determined that reliability of .60 or higher would be acceptable [53].

## Results

All the continuous variables were found to approximate a normal distribution (Kolmogorov-Smirnov Z tests *p *> .10). Results of the reliability analysis are in Table 2. Table 2 shows the means and standard deviations of the 4 testers on the continuous variables, the percentage of findings and percentage of agreement for categorical or ordinal variables, the reliability model used during the analysis, the reliability coefficient with the 95% CI, and the standard error of measurement for continuous variables. Table 3 shows the reliability coefficient values for the overall sample and for each of the two pairs of testers.

## Discussion

Shrout has suggested a classification of reliability coefficients in which values less than 0.10 are considered virtually none agreement; .11 to .40 indicate slight agreement; .41 to .60 indicate fair agreement; values between .61 and .80 indicate moderate; and values greater than .81 indicate substantial agreement [50]. Based on this classification the inter-tester reliability coefficients were substantial for measures of hamstrings length, quadriceps length, gastrocnemius length, soleus length, ITB/TFL complex length, hip abductors strength, and foot pronation. Moderate values of reliability were observed for measures of Q-angle, tibial torsion, hip external rotation strength, lateral retinacular tightness, and test of quality of movement. Measurement of femoral anteversion resulted in fair reliability.

To make valid interpretation of measurements, the measurements must first demonstrate reasonable reliability. Interpretation of the confidence intervals around the values with substantial agreement (above .80) leads to the estimation that the inter-tester reliability of these measures falls anywhere between .68 and .98. Therefore, considering the worst case (lower bound of the 95% CI of hip abduction strength of .68), the reliability of these measures are still satisfactory for clinical use. Measures with a moderate level of reliability had their confidence intervals ranging from .45 and .91, with the lower bound of these intervals ranging from .45 to .58, which warrants some caution when interpreting the findings of Q-angle, tibial torsion, hip external rotation strength, tightness of lateral retinacular structures, and quality of movement. Regarding the interpretation of femoral anteversion, both the reliability coefficient value and the confidence intervals suggest that interpretation of this test's finding may not be consistent.

We have chosen to focus our investigation on inter-tester reliability, rather than intra-tester reliability for two reasons. First, in today healthcare system it is becoming increasingly common to have more than one clinician treating a patient for the same episode of care. Second, data for this study was obtained as part of a larger multicenter study that investigated predictors of function in persons with PFPS. As a result, data were been collected in multiple sites by different clinicians. Furthermore, when designing this study we assumed that the levels of intra-tester reliability would be at least equal or higher than the determined inter-tester reliability.

We are not aware of prior studies that determined the reliability of measuring hamstrings length using the straight leg raise test in a population of patients with PFPS. Our results support the findings in three prior studies and are in conflict with one study. Two studies that were performed with healthy adults and used standard goniometer to measure the straight leg raises reported intersession correlation of r = .88 and an ICC for inter-tester reliability of .99 for this measure [54,55]. Although we acknowledge it may not be appropriate to directly compare results of reliability studies that calculated Pearson correlation coefficient with studies that calculated ICC, such comparison gives us at least an approximation of the consistency of the measurement. Another study with a population of patients with low back pain that used a gravity goniometer to perform the measure reported an ICC of .87 for the inter-tester reliability and a SEM of 6.4 degrees [56]. Our results conflict with the findings of Hunt et al, performed with healthy individuals [57]. They reported fair inter-tester reliability of measuring straight leg raise with an electronic inclinometer, with ICC of .54 and .48 for the left and right leg respectively [57]. Because Hunt, et al, did not provide a description of subject inclusion criteria or a clear description of the test procedure used in their study [57], it is not possible to speculate why their measures were less consistent than our findings or those of other studies. Perhaps the day-long time interval for inter-tester measures used in Hunt et al's study may have been too long and allowed for true variation in tissue compliance over time.

We elected to measure hamstrings length using the straight leg raise test rather than the popliteal angle test to avoid the potential for ceiling effects with the later test [46]. In our clinical experience, the ceiling effect will happen with several patients with PFPS who may completely extend the knee before starting to feel the passive hamstrings resistance during the popliteal angle test. Therefore, in individuals with less hamstrings tightness, the popliteal angle will be limited on the ability to pick up subtle tightness.

Our study yielded better reliability for the patellar tilt test than that reported by Watson et al [24] Watson et al's study included mainly asymptomatic individuals (19 symptomatic and 76 asymptomatic) as subjects and students as testers. They reported inter-tester reliability with Kappa values of .20, .33, and .35 for the three pair of testers, with respective percent agreements of 57%, 47%, and 62% [24]. We believe our study may have had higher reliability because we used experienced therapists who were familiar with the test in clinical practice. Another potential explanation for such difference is the exclusive use of patients diagnosed with PFPS in our study. Having only patients with PFPS may increase the incidence of positive findings and result in a more realistic determination of Kappa values. Watson et al [24] did not report the incidence of positive findings in their study.

Prior studies that used the same method as we did to measure Q-angle have reported lower levels of inter-tester reliability than in our study. Tomsich et al used a sample of healthy young individuals tested by therapists with experience ranging from 2.5 to 5.5 years and reported an ICC of .23 and a SEM of 3.7°[58]. Greene et al had 25 testers measuring each other's knees, two of whom had patellofemoral pain symptoms. They reported inter-tester reliability with ICC values of .20 and .26 for left and right knee respectively [11]. The better reliability in our study could be explained by better standardization of measurements and training of raters, or because all our subjects were diagnosed with PFPS. As increases and decreases in Q-angle are associated with increased patellofemoral pressures, it is possible that patients with PFPS have more variability in the measures of Q-angle than asymptomatic individuals [42]. The decreased data variability in the other studies may have artificially reduced the ICC values. Sutlive et al measured Q-angle on individuals with PFPS in a standing position and reported an ICC of .40 (95% CI: .08; .70) and a SEM of 4.2°[30]. In Sutlive et al's study they do not give details about the methodology of the measure [30]. We have chosen to measure Q-angle in a non functional position to avoid contraction of the quadriceps. Control for quadriceps contraction in a standing position is more difficult than in a supine position. Quadriceps contraction during this measurement could pull the patella sideways and result in inconsistent readings. We believe measuring Q-angle with the participant in a supine position may yield more consistent results.

Our finding indicates a fair to poor reliability of the Craig's test to measure femoral anteversion, which is consistent with prior studies. One study reported Pearson correlation coefficient of r = .47 for inter-tester reliability of this test [15] and another study reported ICC of .17 [30]. The low reliability may be due to the difficulty in accurately palpating the greater trochanter and determining its most lateral position, especially in overweight individuals. To test this hypothesis, we divided the sample according to body mass index (BMI), in which individuals with BMI of .249 or below are classified as normal or underweight, and those with BMI of .25 or above are classified as overweight or obese [59]. The ICC for the 11 individuals with BMI of .249 or below was .81 (95% CI .39; .95), whereas for the 19 individuals with BMI of .25 or above was .20 (95% CI -.30; .60). Therefore, it appears that in overweight individuals measurements of femoral anteversion may be more difficult to perform and consequently less consistent. Until further study investigates the association of BMI and the consistency of femoral anteversion measures we recommend that clinicians make judgments based on the results of this measurement with caution.

Measures of dorsiflexion with the knees extended or flexed at 90° resulted in substantial reliability, which is in disagreement with prior studies. Elvery et al reported ICC of .50 for intertester reliability for ankle passive dorsiflexion [27]. In another study Youdas et al reported an ICC of .28 for measurements of active dorsiflexion [29]. A third study reported ICCs of .29 and .38 for ankle dorsiflexion with knee extended and flexed respectively [30]. We believe our study may have resulted in better reliability for several reasons: 1) We trained the testers to be consistent with positioning the arms of the goniometer; 2) We stabilized the tibia during active dorsiflexion; 3) Measuring active dorsiflexion performed by the subject removes the confounding effect of tester strength that could be a problem if dorsiflexion was measured passively; 4) We used the average of two trials.

Our results are in agreement with previous studies that have indicated good reliability for measures of quadriceps length, hip abduction strength, ITB/TFL complex tightness, and foot pronation. Eng & Pierrynowski have tested the consistency of measures of quadriceps length using the quadriceps femoris muscle angle in a population of female with PFPS and reported an ICC of .94 for intra-tester reliability [60]. A prior study that examined the reliability of measuring hip abduction strength using a hand held dynamometer in runners with iliotibial band syndrome reported substantial inter-tester reliability, with an ICC of 0.96 [61]. Another study used Pearson correlation coefficients to determine test-retest reliability using a hand held dynamometer in two boys with muscular dystrophy and reported correlation coefficients of .86 for hip abduction strength [62]. In a recent study Reese & Bandy tested the reliability of measuring ITB/TFL complex in asymptomatic individuals using the Ober test as a continuous measure as we did and reported an ICC of .90 [63]. Sell et al investigated the reliability of measuring foot pronation using the navicular drop test and reported an ICC value of .73 for the inter-tester reliability [14]. In disagreement with our study and Sell et al study, Sutlive et al reported an ICC of .51 for the navicular drop test [30].

We identified only one study that investigated the reliability of measuring tibial torsion using the same method as we did and they reported an ICC of .32 (95% CI: .07; .53) with a SEM of 6.4° . The better reliability in our study could be explained by better standardization of measurements and training of raters.

Another important point of discussion when comparing our results with results from other studies is that the age of our subjects ranged from 14 to 47 years, which represents a wider range than most of the other studies. Having included adolescents as well as adults formed a heterogeneous sample and potentially created considerable difference between the measurements. Higher variation in the measurement influence the within and between subjects variance, both of which can increase the ICC [49].

To our knowledge this is the first study that reports the reliability of measuring hip external rotation strength and quality of movement in patients with PFPS. Quality of movement was tested during the lateral step down test. This test was developed by our group based on the maladaptive alterations in lower extremity function that are normally observed during physical examination in patients with PFPS [1,4,64,65]. In addition to the step down test being shown to be reliable, we believe it is able to recognize altered movement patterns commonly observed in this population [66]. Further studies should validate this test against referenced measures of function.

When comparing the reliability coefficients calculated with the data from the overall sample with the values obtained from each pair of testers, we observe that the values are consistent for most measurements. Measures that have shown greater differences between both pairs were lateral retinacular length, femoral anteversion, and hip abduction strength. These findings reiterate the above discussion that measures of femoral anteversion are not reliable and that measures of lateral retinacular length warrant some caution in its interpretation. The difference between the two pairs of testers in the measure of hip abduction strength raises additional concerns about this measurement.

An important element of the validity of measurements, and the subsequent ability to accurately interpret these measurements, relies on the evidence of satisfactory reliability and measurement error [67]. Poor reliability and high levels of measurement error reduce the usefulness of a test and limit the extent to which test results can be generalized [67]. Measurement error, determined in this study by calculating the SEM, refers to the hypothetical difference between an examinee's observed score on any particular measurement and the examinee's true score for the procedure [67]. Knowledge of the SEM allows us to put confidence bands around scores and provides a threshold for interpreting the test results over time. Using the SEM of hamstrings length of 4.3 degrees as an example, one can calculate a confidence interval around the obtained score. Let's suppose that the hamstrings length during the straight leg raise test was 80 degrees. If one SEM is added to the hamstrings length measure and one SEM is subtracted from it, an interval is created within which we can be 68% certain that the true measure falls. If two standard errors are added to the measure and two standard errors subtracted from it, a wider interval is created, within which we can be 95% certain that the true measure falls. In our example, if a clinician measures hamstrings length 80 degrees and the SEM is 4.3 degrees, we can be 68% certain that the true hamstrings length is between 75.7 and 84.3 degrees and 95% certain that it is between 71.3 and 88.6 degrees. When interpreting changes over time, if the measure changes from 80 to 84.3 degrees from one occasion to the next, one can be 68% confident that true change has occurred, if the measure changes from 80 to 88.6 degrees, the level of confidence in such change increases to 95%. Further validation might be gained in future studies that determine how responsive to change these measurements are following interventions.

There is currently no consensus regarding the number of SEMs an individual's score must change for that change to confidently exceed measurement error. In other words, there is no agreement about what is the preferred level of confidence. Previous researchers have reported one SEM as the best measure of meaningful change on health-related quality of life measures [52]. The number of SEMs that would reflect meaningful change on measures of physical impairments is not known. Moreover, the SEM has several properties that make it an attractive statistic for determining clinically meaningful change. First, the SEM accounts for the possibility that some of the change observed with a particular measure may be attributable to random error. Secondly, the SEM is independent of the sample under investigation; that is, the SEM is expected to remain relatively constant for all samples taken from a given population. Third, the SEM is expressed in the original metric of the measure, aiding its interpretation [52].

One limitation of this study is that the reliability results found may be an over-estimate compared to real clinical practice. Many factors may have influenced the measurements collected during this research. The experimental environment may have been unrepresentative of measures taken in a busy clinic. Specific aspects of clinical practice may lower reliabilities values of the measures investigated in the present study. The testers in this study were well trained to perform the measures and followed a standardized protocol. In the real clinic, clinicians work under time restraints and may follow a less strict set of rules when testing their patients. Furthermore, several variations in technique may exist across clinicians. Even those who are very accurate in the use of the tests may never have had the opportunity to standardize their own techniques with those of colleagues. We believe the information about the reliability of the measures investigated in this study may be of clinical relevance if the clinicians who intend to use such measures are rigorous in the use of the tests as here described and if they make the effort to standardize the technique with the colleagues.

To validate the use of the measures of impairments associated with PFPS tested in this study, further research is warranted in a number of areas. It should be determined whether these impairment measurements are related to pain and function in individuals with PFPS. It should also be determined whether changes in these impairment measurements will be associated with improvement of pain and function after completing a rehabilitation program.

## Conclusion

Several of the impairments associated with PFPS had good reliability. Inter-tester reliability coefficients were substantial for measures of hamstrings length, quadriceps length, plantar flexors length, ITB/TFL complex length, hip abductors strength, and foot pronation, which ensure valid interpretation of these tests results in clinical practice. Moderate values of reliability were observed for measures of Q-angle, tibial torsion, hip external rotation strength, lateral retinacular tightness, and test of quality of movement, which warrants some caution when interpreting the findings of these tests. Measurement of femoral anteversion resulted in fair reliability, suggesting that interpretation of this test may not be consistent. Additional evidence is needed to support their use by testing if these impairment measurements are related to physical function and whether or not they can be used to guide treatment planning which ultimately would result in successful treatment outcomes.

## Competing interests

The author(s) declare that they have no competing interests.

## Authors' contributions

SRP conceived and coordinated the study, performed statistical analysis, and drafted the manuscript. KF, JJI, and JDC participated in the study design and revision of manuscript. SJ, BRH, and DAB have acquired the data and were involved in drafting the manuscript. All authors read and approved the final manuscript.

## Pre-publication history

The pre-publication history for this paper can be accessed here:



## Figures and Tables

**Table 1:** Demographic characteristics of the sample. Values represent the mean (Standard Deviation) unless otherwise stated.

Variable (n= 30)	
Age in years	29.1(8.4)
Number of subjects in each age range:	
From 14 to 19 years	2 (7%)
From 20 to 29 years	16 (53%)
From 30 to 39 years	7 (23%)
From 40 to 47 years	5 (17%)
Number of females	17 (59 %)
Height in cm	171 (11.1)
Weight in kg	79 (18.6)
Body Mass Index as kg/cm^2^	.26 (.05)
Numeric Pain Rating Scale* score	3.9 (1.9)
Activity of Daily Living Scale** score	67.3 (17.3)

* Numeric pain rating scale ranges from 0 (No Pain) to 10 (Worst Imaginable Pain) points.

** Activity of daily living scale ranges from 0 to 100 points. One hundred indicates absence of symptoms and functional limitations.

**Table 2:** Means and standard deviations of the 4 testers of the continuous variables, percentage of findings for categorical or ordinal variables, the reliability model used during the analysis, the reliability coefficient, the percentage of agreement for Kappa calculation, the 95% CI, and the standard error of measurement for continuous variables.

Variable (n = 30)	Mean (SD) or % of findings	Model used	Reliability coefficient and % of agreement for Kappa calculation	95% CI	SEM
Hamstrings length (degrees)	81.5 (15.0)	ICC (2, 2)	.92	(.82; .96)	4.3
Lateral retinacular length (tight, normal)	83% tight	Kappa	.71 (93%)	(.57; .86)	--
Q-angle (degrees)	12.2 (4.3)	ICC (2, 1)	.70	(.46; .85)	2.4
Tibial torsion (degrees)	17.6 (5.4)	ICC (2, 1)	.70	(.45; .85)	2.9
Quadriceps length (degrees)	138.5 (12.3)	ICC (2, 2)	.91	(.80; .96)	3.8
Femoral anteversion (degrees)	12.8 (6.1)	ICC (2, 1)	.45	(.10; .70)	4.5
Gastrocnemius length (degrees)	9.3 (5.8)	ICC (2, 2)	.92	(.83; .96)	1.6
Soleus length (degrees)	16.0 (6.0)	ICC (2, 2)	.86	(.71; .94)	2.2
Hip external rotation strength (Kg)	17.1 (5.2)	ICC (2, 2)	.79	(.56; .91)	2.4
Hip abduction strength (Kg)	12.9 (4.6)	ICC (2,2)	.85	(.68; .93)	1.8
ITB/TFL complex length (degrees)	15.5 (11.1)	ICC (2, 2)	.97	(.93; .98)	2.1
Foot pronation (mm)	5.9 (2.7)	ICC (2, 1)	.93	(.84; .97)	0.7
Quality of movement (from 0 to 1 = good; from 2 to 3 = medium; 4 and above = poor)	33% good 50% medium 17% poor	Kappa	.67 (80%)	(.58; .76)	--

**Table 3:** Comparison of reliability coefficient for the overall sample and for each of the pairs of testers.

Variable	Reliability coefficient		
	
	Overall sample (n = 30)	Pair 1 (n = 20)	Pair 2 (n = 10)
Hamstrings length (degrees)	.92	.88	.98
Lateral retinacular length (tight, normal)	.71	1.0	.55
Q-angle (degrees)	.70	.56	.77
Tibial torsion (degrees)	.70	.60	.83
Quadriceps length (degrees)	.91	.90	.93
Femoral anteversion (degrees)	.45	.31	.64
Gastrocnemius length (degrees)	.92	.78	.97
Soleus length (degrees)	.86	.85	.92
Hip external rotation strength (Kg)	.79	.71	.97
Hip abduction strength (Kg)	.85	.86	.59
ITB/TFL complex length (degrees)	.97	.90	.99
Foot pronation (mm)	.93	.89	.95
Quality of movement (from 0 to 1 = good; from 2 to 3 = medium; 4 and above = poor)	.67	.62	.67
